# Kirigami-Triggered Spoof Plasmonic Interconnects for Radiofrequency Elastronics

**DOI:** 10.34133/research.0367

**Published:** 2024-05-01

**Authors:** Xincheng Yao, Min Li, Shuchang He, Liqiao Jing, Chenming Li, Jie Tao, Xiaonan Hui, Fei Gao, Jizhou Song, Hongsheng Chen, Zuojia Wang

**Affiliations:** ^1^ State Key Laboratory of Extreme Photonics and Instrumentation, ZJU-Hangzhou Global Scientific and Technological Innovation Center, Zhejiang University, Hangzhou 310027, China.; ^2^ International Joint Innovation Center, Key Lab. of Advanced Micro/Nano Electronic Devices & Smart Systems of Zhejiang, The Electromagnetics Academy at Zhejiang University, Zhejiang University, Haining 314400, China.; ^3^ Department of Engineering Mechanics, Key Laboratory of Soft Machines and Smart Devices of Zhejiang Province, State Key Laboratory of Brain-Machine Intelligence, Zhejiang University, Hangzhou 310027, China.

## Abstract

The flexible and conformal interconnects for electronic systems as a potential signal transmission device have great prospects in body-worn or wearable applications. High-efficiency wave propagation and conformal structure deformation around human body at radio communication are still confronted with huge challenges due to the lack of methods to control the wave propagation and achieve the deformable structure simultaneously. Here, inspired by the kirigami technology, a new paradigm to construct spoof plasmonic interconnects (SPIs) that support radiofrequency (RF) surface plasmonic transmission is proposed, together with high elasticity, strong robustness, and multifunction performance. Leveraging the strong field-confinement characteristic of spoof surface plasmons polaritons, the Type-I SPI opens its high-efficiency transmission band after stretching from a simply connected metallic surface. Meanwhile, the broadband transmission of the kirigami-based SPI exhibits strong robustness and excellent stability undergoing complex deformations, i.e., bending, twisting, and stretching. In addition, the prepared Type-II SPI consisting of 2 different subunit cells can achieve band-stop transmission characteristics, with its center frequency dynamically tunable by stretching the buckled structure. Experimental measurements verify the on-off switching performance in kirigami interconnects triggered by stretching. Overcoming the mechanical limitation of rigid structure with kirigami technology, the designer SPIs exhibit high stretchability through out-of-plane structure deformation. Such kirigami-based interconnects can improve the elastic functionality of wearable RF electronics and offer high compatibility to large body motion in future body network systems.

## Introduction

A body area network (bodyNET) enables wireless transmission of various human body physiological signals to the digital world, allowing extension of individual perception abilities and remote healthcare [1]. A complete bodyNET is envisioned to be a secure and energy-efficient networking that can monitor physiological signals by flexible sensor nodes [2], transmit collected health data through on-body interconnection channels [3], analyze individual health status by means of artificial intelligence or cloud computing [4], and offer medical advice before clinicians perceive symptoms [5]. Emerging flexible sensor techniques, such as nanomesh receptor [6], fluid passage [7], detuned radiofrequency (RF) identification tag [8], and integrated electrochromic display [9], have enabled real-time monitoring of multiple physiological signals and improved mechanical compatibility to human skins. The interconnection of individual sensors to an on-body network similar to wireless communication of distributed systems is, however, still challenging due to being energy-consuming and vulnerable to interception of surrounding conditions. Conventional conductive threads that connect sensor nodes suffer from rigid-to-soft incompatibility and low-frequency transmission restriction [10–11]. Electronic fibers [12–13] or textiles [14] fused with bioelectronic components are capable of delivering electronic signals among sensors while maintaining superior breathability and comfort, but the conductivities of which are usually several orders of magnitudes smaller than conventional metals [15], hampering their application in RF interconnections because of high ohmic losses.

Metasurface textiles have recently emerged as a promising platform for on-body RF interconnection [16]. Metasurfaces [17–20] can arbitrarily manipulate the propagation and scattering of on-body electromagnetic waves and thus be used to suppress the reflection and absorption of the human body with improved energy efficiency. Based on the textiles technology, a near-field communication system made out of discrete magneto-inductive elements and attached on clothing with a heat press vinyl has been introduced, achieving bound magnetic surface-wave propagation below gigahertz frequencies [21]. On the other hand, although existing radiative and highly redirected propagation of RF signal along the body surface shows the wireless signal transmission ability between sensor nodes through the special textile antenna [22–26], it does not solve the obstruction of wave propagation around the body. By integrating the wave-propagation structure into clothing and placing the conductive textiles near the body, this strategy can potentially avoid obstruction around surrounding space and therefore achieve highly efficient and point-to-point interconnect communication [27–28]. Due to the rigid feature of conductive materials and rigid-to-soft incompatibility, previous textile interconnects at RFs are severely sensitive to large-scale stretching. Therefore, elastronic materials and interconnects that possess strong robustness and versatile electronic functionalities against geometrically stretching are crucial and urgently demanded in high-compatibility bodyNETs for at-will body motions.

**Fig. 1.**
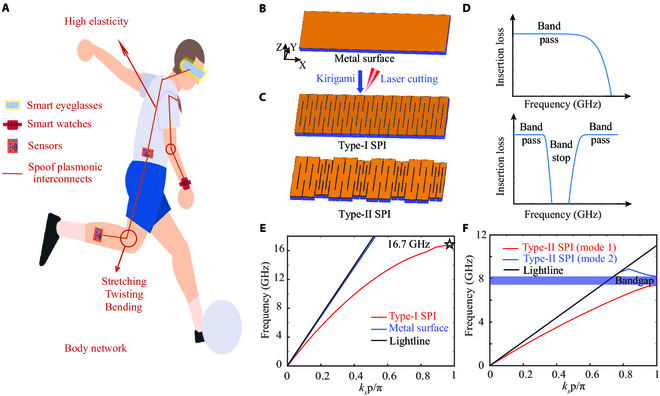
Wireless bodyNETs based on high-elasticity SPI. (A) Conceptual illustration of the body network based on SPIs. The schematic illustration (B) before and (C) after laser cutting metal surface. (D) Illustrated insertion loss vs. frequency for these 2 stretchable SPIs in (C). (E) Dispersion curves of stretchable Type-I SPI and metal surface. (F) Mode 1 (fundamental mode) and mode 2 (second mode) for the stretchable Type-II SPI. The corresponding cutoff frequency is 16.7 GHz.

In this article, we proposed a new paradigm to construct high-elasticity and reliable spoof plasmonic interconnects (SPIs) that support RF surface wave transmission toward bodyNET (Fig. 1A). In contrast to previous studied stretchable elastronic materials, which usually adopt an elastic substrate with a relatively rigid metallic structure [29], our approach employs kirigami-based metasurface that possesses high stretchability and strong robustness and enables extraordinary manipulation over the elastic waves [30], brainwaves [31], and RF field [32–34]. The kirigami slits between 2 ribbons are preprocessed by laser cutting the metal surface with minimized additional manufacturing waste. This kirigami pattern can be arbitrarily designed, constructed, and expanded at will to satisfy the demands of human body on comfort and breathability. Inspired by the strong field-confined electromagnetic waves on metallic surface [35–36], we developed novel deformable multifunction interconnects that achieve high-efficiency transmission performance after stretching metal surface structure. Unlike conventional planar corrugated grooves [37–41], our design showcases a higher degree of freedom and flexibility due to geometric transformation from 2-dimensional (2D) to 3D structures via kirigami technology. Importantly, the strong robustness and excellent stability during various deformations, including bending, twisting, and stretching, can be well maintained for the broadband and band-stop transmission of the kirigami-based Type-I and Type-II SPIs, respectively. In addition, the center frequency of band-stop transmission can be dynamically tuned by stretching the buckled structure. Experimental measurements verify the high-efficiency and secure wireless signal transmission of broadband and band stop along the body surface. Meanwhile, the mechanical response of kirigami-based interconnects with high elasticity guarantees the requirement on daily motion wear toward metasurface textiles applications. Overall, the simple manufacturing strategy, high-efficiency transmission, strong robustness, and multifunction of the proposed deformable SPIs promise an alternative avenue toward elastronic devices in bodyNET systems.

## Results and Discussion

Wearable electronic textiles detecting and recording physiological signals and sports conditions can be used to construct a network of smart eyeglasses, smartwatches, and sensors around the body known as the bodyNET. The bodyNET structured with SPI supporting surface plasmonic modes and thus propagating RF waves around the body is schematically illustrated in Fig. 1A. The proposed SPI shows high elasticity during body movement and reliability under several types of deformation, i.e., stretching, twisting, and bending. Firstly, we consider a metal surface supporting Zenneck waves along the interface as the initial state [42], which is composed of a simply connected metallic surface and a dielectric substrate (see Fig. 1B). A schematic illustration of kirigami-based SPI is exhibited in Fig. 1C. The unstretched Type-I and Type-II SPIs with the “ribbon” patterns of parallel slit cut are accomplished through laser cutting on the metal surface. In this process, the high intense and precision laser beam (15-W femtosecond laser and 0.1-mm kerf width) strikes the cutting position with the help of its intense heat. Additionally, the simplified pattern and laser cutting marking speed of 900 μm/s can minimize heat-impacted zone and avoid the deformation of materials. Therefore, this subtractive manufacturing approach for the kirigami-based interconnects shows the potential for mass production and quick process.

According to the predesigned kirigami patterns, the stretchable Type-I and Type-II SPIs achieve multifunctional transmission characteristics including broadband and band stop, which exhibit immense prospects for constructing functional microwave or terahertz circuitry worn on the body and digital information systems in the future [43–44]. Insertion loss of 2 proposed stretchable SPIs at various frequencies are illustrated in Fig. 1D. In order to more comprehensively understand the transmission performance of the interconnects, dispersion curves analysis of the SPI cell has been presented using the eigenmode solver of CST Microwave Studio. The asymptotic dispersion curve of the Type-I SPI with stretchable values up to 100% deviates slowly and maintains below the light line with the increase of *k_x_p*/*π*, as shown in Fig. 1E. Comparing with the Zenneck waves on the metal surface, the stretchable Type-I SPI supporting surface plasmonic modes showcases the ability of strong field confinement and the characteristic of slow wave. According to the spoof surface plasmonic polaritons theory [28,38], the surface plasmonic dispersion can be artificially tuned through optimization on the geometrical parameters, i.e., facet B length *l*_b_ and ribbon widths *w* (Fig. S1). High-efficiency broadband transmission along an optimized stretchable SPI is then achieved. To confirm the surface plasmon-like nature of the modes, field distribution and normalized exponential field decay along the middle white line on the *y*-*z* plane have been studied (Fig. S4). The spatial electric field is confined within a few millimeters in the vicinity of the interface (body surface), which is beneficial for integration in wearability clothing. Furthermore, the dispersion curves of the Type-II SPI at the same stretchable value are investigated, and an obvious bandgap prohibiting energy propagation inside from 7.4 to 8.2 GHz can be observed between the passbands of mode 1 and mode 2 (see Fig. 1F). Similarly, the impact of geometric parameters (*w*_1_, *w*_2_, and *l*_b_) on the dispersion curves of the Type-II SPI has been investigated (Figs. S2 and S3). The band-stop bandwidth is highly sensitive to *w*_1_ and *w*_2_ yet much less to *l*_b_. This provides a theoretical guidance on the tuning of the stopping band of Type-II SPI by geometric parameters.

**Fig. 2.**
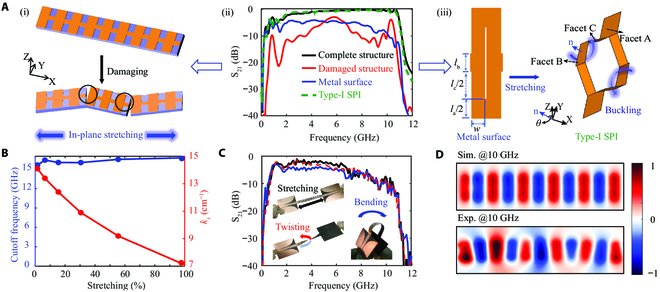
Robust transmission performance of high-elasticity Type-I SPI. (A) (i) Illustration before and after damaging corrugated groove structure; (ii) transmission comparison; (iii) schematic configuration before and after stretching Type-I SPI. The blue arrow (n) indicates the normal vector perpendicular to the facet B. (B) Dispersion curves analysis for the Type-I SPI with different stretchable states. (C) Measured transmission and experimental photograph of the SPI with different deformable states. (D) Simulated (upper) and measured (bottom) *z*-component near-electric field distributions of the Type-I SPI at 10 GHz.

Figure 2A shows the comparison of transmission performance before and after stretching of the rigid planar corrugated structure and SPIs. The tear opening angle of the damaged structure is 5°, as shown in Fig. 2A (i). It can be observed that transmission efficiency experiences severe degeneration when slight mechanical damage is introduced on the rigid metal (Fig. 2A, ii). The proposed SPI provides a versatile platform for manipulating RF wave propagation and achieving high-elasticity deformable structure and further improves the transmission performance of wireless signals between device nodes worn on human body. To validate the effectiveness of the design strategy, we have simulated and measured the electromagnetic performance and field distribution of the Type-I SPI at various deformable states. Figure 2A (iii) displays the configuration illustration of the laser-cutting metal surface and stretchable Type-I SPI. To simplify the deformable process, the “virtue folds” at critical locations that exhibit the most obviously bent deformation and concentrated stress are introduced [45], with the assumption of rigid facets in-between folds (facets A, B, and C). The dihedral angle *θ* between facet B within the Type-I SPI cell and the *x*-*y* reference plane of the global Cartesian coordinate represents the deformation degree of stretching. More information about the relationship between *θ* and the stretchable displacement is shown in Supplementary Materials Section S3. Indeed, based on the designed crease, these folds can naturally take place when in-plane stretching is strong enough to achieve the desired out-of-plane buckling deformation. The transmission of the Type-I SPI with a stretchable value of up to 100% surpasses that of Zenneck wave transmitted on the metal surface (Fig. 2A, ii). Here, the metal surface and Type-I SPI structure have the same physical length (158.4 mm).

The dispersion relationships analysis of the Type-I SPI with different stretchable values are summarized in Fig. 2B, where *k_x_* represents the wave vector of the proposed unit cell along the propagating direction. With external mechanical stimuli, the interconnect could be stretched sustainably and flexibly, leading to periodic change and further dynamical control over the momentum of the unit cell. Although the periodicity of the designed structure increases rapidly with stretching, it is worth noting that the cutoff frequency of different stretchable shapes is almost invariableness at around 16 GHz. Meanwhile, *k_x_* of the Type-I SPI along the *x* direction is reduced from 14.1 to 7.1 cm^–1^, corresponding to an increased stretching from 1.5% to 100%. It is stressed that the cutoff frequency of the SPI determined by the corrugated grooves from hybrid plasmonic waveguide as double-side excitation sources of the interconnect is about 11.4 GHz, instead of that (16 GHz) of the designed SPI (more details in Supplementary Materials Section S4). The dispersion curves and corresponding unit cell of the corrugated grooves show the asymptotic frequency of 11.6 GHz, which is consistent with the cutoff frequency (Fig. S7A and B). The near-field *E*_z_ distribution of the overall structure at 12 GHz has been further investigated to confirm the suppressed energy transfer from the corrugated grooves (see Fig. S7C). Moreover, the transmission performances of the Type-I SPI with stretchable values up to 1.5%, 6.4%, 15.5%, 30.5%, 55.6%, and 100% are also demonstrated, showing high stability and strong robustness against stretching (Fig. S8). For the SPI with a stretchable value up to 15.5%, the 3-dB bandwidths range from 1.3 to 10.8 GHz, meaning the high-efficiency and broadband transmission of the proposed interconnect. The measured return loss is lower than −10 dB from 1.3 to 10.7 GHz, indicating excellent mode conversion, matched impedance, and low-loss transmission between the stretchable Type-I SPI and conventional coplanar waveguides (50 Ω impedance) [38].

Reliability is one of much importance for high-elasticity and stretchable SPI to accommodate realistically complex deformation near the body surface. Experiments and simulations have been performed to verify the robust and stable transmission characteristics of the deformable Type-I SPI. The simulated results exhibit a slightly degraded transmission efficiency (S_21_) under bending and twisting, without compromising their functionality as shown in Fig. S9. In addition, the finite element analysis simulation of the stretchable SPIs is also performed to prevent structure damage during practical deformation. For the deformable SPI with bending and twisting, it is observed that the maximum principal strain is about 5.8% and 2.6% at the tips of the slit cuts, respectively, which is less than the damage limit (6%), indicating no breakage in the interconnect as shown in Fig. S10 (more detail about the bending and twisting is shown in Supplementary Materials Section S5). Figure 2C shows the photos of the experimental setup and the measured results of the interconnects. The fabricated devices maintain stable transmission performance under 180° bending and 180° twisting, which is consistent with the simulation results. To more deeply understand the special field distribution of spoof surface plasmonic polaritons, the *z*-component near-electric field distributions of the Type-I SPI have been simulated and measured, showing the high-efficiency propagation along the SPI at an in-band frequency point of 10 GHz (Fig. 2D). The corresponding normalized amplitude profiles along the middle line (*y* = 0) of the spatial field distribution are also presented in Fig. S11. It can be observed that the amplitude of the received waveform has very little attenuation after passing through the stretchable SPI.

The effects of body temperature and applied pressure on the transmission performance of Type-I SPI are evaluated. There is no obvious change in the transmission of the stretchable SPI after the temperature from 36 to 40 °C and applied high pressure of 11.5 and 57.7 kPa as shown in Fig. S12. Furthermore, the endurance measurement of the interconnect is performed to ensure stable RF transmission after multiple stretching. As shown in Fig. S13, the RF transmission of the SPI is stable and repeatable over cycling in-plane stretching from 0% to 15% at an external load, with an almost negligible decrease in the signal transmission over 100 cycles. Such results ensure that the designed stretchable SPI structure holds a very promising future in the bodyNET and wearable devices at RFs. More importantly, this kirigami-based strategy can be easily bended, twisted, and stretched, indicating outstanding compliance to high-elasticity deformation and adaptation to various textiles [46].

**Fig. 3.**
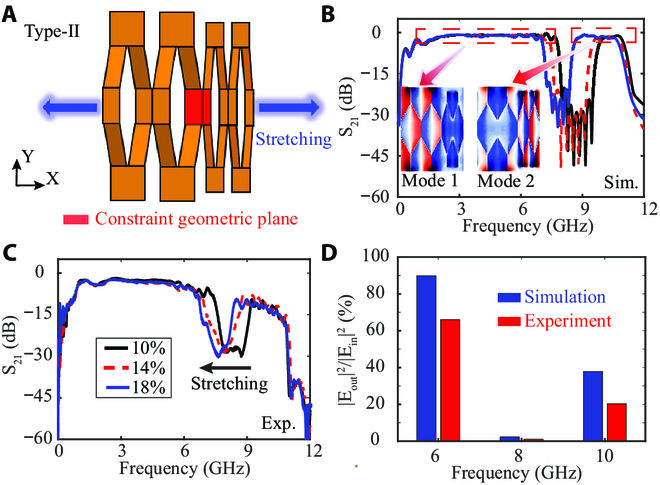
Transmission performance of the Type-II SPI with tunable band stop. (A) Schematic configuration of proposed Type-II SPI. (B) Simulated and (C) measured transmission of the Type-II SPI with different stretching states. Inset: *E*_z_ field distributions of the mode 1 and mode 2. (D) Energy transfer histograms of z-component near-electric field distributions (on the plane 3 mm over the deformable Type-II SPI) of the Type-II SPI at 6, 8, and 10 GHz.

In some special scenarios, electromagnetic band-stop structures providing an important function between the wearable sensors and/or antennas can be used to prevent undesirable spectra response and noise interference. The electromagnetic performances of Type-II SPI with a robust band-stop feature are illustrated in Fig. 3. The supercell of the stretchable Type-II SPI containing 2 cells with different geometry and the individual unit (see Fig. 2A and Fig. S5C) now become a combination of large and small subcells, as shown in Fig. 3A. During the stretching of the combined unit cells, we assume a critical constraint regarding the direction of facet rotation based on the rigid facet: the adjacent facets’ tilted direction and rotation angle must be the same at the boundary between 2 different subcells for geometric deformation. That is, the interconnects here can be stretched by external stimulation, causing the marked out-of-plane deformation of both different kirigami sheets. As a result, the stretchable and buckled SPI is applied to generate dynamically tunable 1-dimensional periodic structures.

The transmissions S_21_ of the band-stop Type-II SPI with stretchable values up to 10%, 14%, and 18% are simulated (Fig. 3B). One can clearly see that the central frequency of the band stop continuously redshifts as the stretching value increases. The stretchable kirigami SPI is a simple and effective approach for achieving and controlling for wave propagation, and the correlation between stretching periodicity and tunable band stop is firmly established [30]. In order to provide a more intuitive and comprehensive understanding of the fundamental (mode 1) and second-order (mode 2) modes of the proposed Type-II SPI, the electric field distributions of the 2 modes at *z* = 0 are shown (the inset of Fig. 3B). It can be seen that both mode 1 and mode 2 are the even modes with symmetric field patterns, indicating efficient signal propagation and energy transfer of the 2 modes along the interconnects. Therefore, the dispersion curves of Type-II SPI mode 1 and mode 2 in Fig. 1F correspond to symmetric field modes and imply the bandgap induced by the difference of the effective refraction index of the neighboring subcells structures [47–48]. These results regarding field distribution together with the asymptotic frequencies can be used to confirm the propagating mode and band-stop feature supported in the proposed Type-II SPI. Moreover, to experimentally evaluate the electromagnetic performance of Type-II SPI, the measured transmissions for the proposed structure are presented in Fig. 3C. Good agreement confirms the ability of tunable frequency with varied stretching and achieves real-time dynamic control for the band stop. It is noted that the machining tolerance and nonuniformity of the fabricated deformable samples result in the deteriorated S_21_ at the high-frequency band while having a slight influence on it at the low-frequency band.

The energy transfer of the stretchable Type-II SPI at different frequencies (i.e., 6, 8, and 10 GHz) have been demonstrated in Fig. 3D. Electromagnetic signals along the interconnects are blocked in the stop band (8 GHz), while it is delivered in the pass band (6 and 10 GHz), which is consistent with the dispersion predictions. Meanwhile, the near-field analyses and corresponding normalized amplitude profiles along the middle line (*y* = 0) of the spatial field distributions at 6, 8, and 10 GHz are also exhibited (Figs. S14 and S15). It is interesting that electromagnetic energy transfer at 8 GHz is prohibited from going through the Type-II SPI and decays exponentially (see the envelope in Fig. S15B and E) toward the *x* direction, as indicated by the corresponding reduced S_21_ parameter, which can be attributed to the difference of the effective refraction index between 2 subcells structures. The experimental results of the near-field distributions are measured by a near-field scanning system (as presented in Fig. S17).

**Fig. 4.**
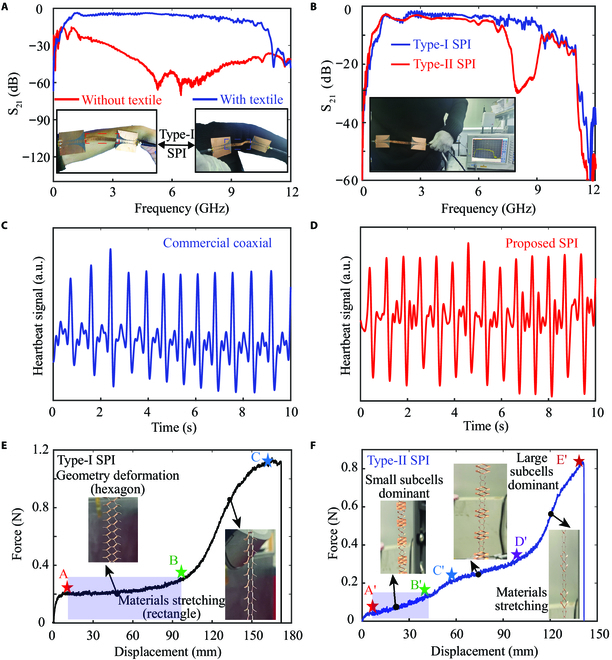
Experimental verification on on-body wireless signal transmission and mechanical performance of the SPIs. (A) The transmissions and corresponding experimental photos of Type-I SPI with and without textiles worn on arm part. (B) The experimental photos and corresponding transmissions of Type-I and Type-II SPIs worn on belly part of the body. Extracted heartbeat signals of the Tx/Rx pair antenna connecting the (C) commercial coaxial and (D) proposed SPI within 10 s. Force-displacement response and photographs corresponding to different curves process of (E) Type-I and (F) Type-II SPIs.

To evaluate the wireless transmission ability between wearable devices on the body, we have measured the transmissions of the kirigami-based SPIs near the body position and investigated their elasticity and robustness performance. When the Type-I SPI with textiles connecting double-side hybrid plasmonic waveguides is placed near the arm, the signal can propagate through the stretchable period structure, as shown in Fig. 4A. This contactless textile results in a much higher efficiency signal transmission in comparison with the model without textile, which avoids the RF wave scattering and absorption by biological tissues in the body. In particular, the confinement of energy/signal onto the metasurface’ textile ensures data security and energy-efficient transmission between wearable device nodes on human body [28]. The packaging of the interconnects plays a key role in the actual wearable devices. The plasmonic interconnect integrated with textiles as a packaging step can be achieved to provide wireless networking ability in the health monitoring. Based on the metasurface’s textile design, we simulated the transmission performance of corresponding textile SPI with a stretchable value up to 15.5% (see Fig. S16). Here, the simplified model composed by the textile and metal was designed. The transmission of the SPI with and without textile packaging has no obvious change. With the rapid development in conductive textile production techniques in the industry [49], more textile-based networking methods can be utilized to integrate the interconnect with flexible fabric. Furthermore, we evaluate the robustness of the transmission performance during stretching Type-I interconnects, and real-time monitoring of the signal transmission is performed with a hybrid plasmonic waveguide connecting the Vector Network Analyzer via SubMiniature version A connectors (see Movie S1). By combining the large and small subcells to achieve the band-stop transmission functionality of surface plasmonic modes, we arrange 2 designed SPIs on the body. Though the stretching interconnect is vulnerable to the body and complex deformation (Fig. 4B), the S_21_ parameters of both them can be well maintained due to their high field confinement and mechanical robustness. The degraded transmission performance of the Type-II SPI results from the nonuniform deformation and machining tolerance. Importantly, the Type-II SPI close to the body has the ability to keep the band-stop functionality and highly efficient transmission in the pass bands. In addition, the bandgap frequencies can be dynamically tuned by simply tailoring the periodic global structure (Movie S2). Here, it is difficult for rigid dielectric materials or metal to go back to their original state owing to the local plastic deformation. Despite this is difficulties, our theoretical method of kirigami-induced spoof plasmonic modes is general and can be flexibly extended to other materials. We envision that these plastic deformations can be avoided by optimizing the cut tip shape or selecting flexible materials [30,50], further achieving recoverable original state and frequency blueshift.

Physiological signs monitoring plays an important role in evaluating health status and body motion conditions. To assess the accurate transmission ability of physiological biosignals, we have conducted an additional experiment based on near-field cardiac sensing technique to demonstrate the capability in heart rate detection, as shown in Fig. 4C and D. The experimental setup is illustrated in Fig. S18, where software-defined radio (B210) is connected between SPI and computer. A Tx/Rx pair antenna (2.4 GHz) near the left chest continuously detects the mechanical vibration signal from local heartbeat motion [51–52]. Then, the signal received by the antenna is transmitted to the host computer through the commercial coaxial and our proposed interconnects, respectively. It can be seen that the extracted heartbeat signal of the subject shows periodic cardiac activity within 10 s, which further verifies the efficient propagation of the RF signal compared with the commercial coaxial. During the testing, the subject stays stable and relax on a chair to ensure a stable heart rate. The subject’s heart rate is estimated as 81 and 86 bpm for the commercial and SPI plasmonic interconnects, which is within the normal range.

Comparisons with previous soft interconnects in terms of materials, stretchability, functionality, mechanism and frequency are listed in Table. The designer SPIs reported here possess a dual function, i.e., bandpass and band stop, which enable it to construct signal-processing and computational circuit systems. Buckling-engineering-enabled multifunctional interconnects via kirigami technology deliver competitive stretchability although its structure is of rigid-to-soft incompatibility (metal/dielectric). Besides, it is worth pointing out that the out-of-plane buckling 3D SPIs can preserve RF surface-wave propagation ability from conventional planar groove structures. In general, our designed SPIs are the first kirigami-based RF interconnects. If considering further optimization of the interconnect including materials chemistry, pattered design, and incorporation of additional textile fabrication schemes, there must be a great chance to improve the comprehensive performance of this new type of interconnect.

**Table.** Performance comparisons of soft interconnects

Materials	Stretchability	Functionality	Mechanism	Frequency	References
Metal/dielectric	No	Bandpass	Spoof plasmonics	10–11 GHz	[40]
Conductive textiles/clothing	No	Bandpass	Spoof plasmonics	2.4–2.5 GHz	[27–28]
Metal/elastomer	Yes	Bandpass	Spoof plasmonics	2–7 GHz	[29]
Metal/textiles	No	Bandpass	Magneto-inductive waves	13–14 MHz	[21]
Fibers electronic	Yes	/	Electrical signal	MHz	[10,55,56]
Kirigami-based liquid metal/elastic substrate	Yes	/	Electrical signal	8–12 Hz	[31]
Kirigami-based metal/dielectric	Yes	Bandpass/band stop	Spoof plasmonics	1.3–10.8 GHz	This work

The mechanical responses of the SPI at different stretchable values have been characterized using the uniaxial test machine as shown in Fig. 4E and F. The effect dominated by the stiffness properties of materials and geometry deformation on the force-displacement curves are investigated. As predicted in previous studies [53], the mechanical response of those kirigami-based structures usually exhibits 3 distinct regions including initial elastic response, plateau stress, and final stiffness. Here, we start by focusing on a Type-I kirigami-based SPI comprising 36 units. The force-displacement curves with controllable and nonlinear features and corresponding experimental process photos are illustrated in Fig. 4E. The initial response for the proposed structure is a plateau stress generated by the out-of-plane buckling of the ribbon (facet B) corresponding to the curves from A to B points after the linear elastic response of the in-plane stretching. At this stage, a large displacement arises from geometry deformation, and thereafter, a 3D cubic structure is formed. Then, the applied force rises rapidly when the stretchable displacement approaches the critical value (i.e., geometry structure from hexagon to rectangle), which is caused by the stretching of material until the structure is destroyed. However, the force-displacement curves of the Type-II SPI emerging 2 stress plateaus (from A′ to B′ and C′ to D′) show a noticeable difference from that of the Type-I one, as shown in Fig. 4F. For the first stage, due to the difference in material stiffness between small and large subcells, the relatively large displacement caused by the geometry deformation of the small unit cell is dominant. As the applied strain increases (for the second stress plateau), the deformable effect of the large unit cell on stretchable displacement is more important, and then the material stretching induces a sharp increase from D′ to E′ points. The corresponding experimental photos are exhibited in inset of Fig. 4F and Fig. S19. It is noteworthy that our designed SPI structures focus on the mechanical properties of the first stage (i.e., A to B and A′ to B′), which is highlighted in blue shadow.

## Conclusion

In summary, we propose high-elasticity, strong robustness, and multifunctional kirigami-based SPIs by laser cutting designer patterns on a simply connected 2D metallic surface and subsequently stretching predesigned ribbon sheets to achieve 3D architectures. The stretchable Type-I interconnects supporting the surface plasmonic modes at RF bands have been demonstrated, which can maintain excellent transmission performance, strong field confinement, and stability under high stretchability from 1.5% to 100%. The deformable SPI can support the robust propagation of wireless signals, against 180° bending and 180° twisting, and continues stretching. Furthermore, by elaborately designing the unit cells, the Type-II SPI with the band-stop performance caused by the tunable bandgap between mode 1 and mode 2 is developed. Owing to the varied periodicities in kirigami-based deformation, the band-stop center frequency can be dynamically redshifted when the proposed SPI is stretched from 10% to 18%. The proposed SPIs can achieve reliable and stable signal transmission performance. Furthermore, we have also demonstrated the potential of using textiles to engineer broadband and band-stop surface plasmonic mode propagation of high-elasticity interconnects on textiles. Mechanical response of the interconnects indicates that kirigami interconnects are a versatile platform to achieve high stretchability. With the ongoing development of body-worn or wearable electronic devices, the proposed kirigami multifunctional SPIs that show better elastic behaviors can be useful in a variety of applications in wireless communication, health monitoring, and biomedical fields.

## Materials and Methods

### Device fabrication

A copper metal layer is firstly printed on the top surface of an ultrathin dielectric substrate (Rogers RT5880, the relative permittivity is 2.2 and thickness is 0.127 mm) using the standard printing circuit board technology, and then the elastic Type-I and Type-II SPIs with a “ribbon” designed patterns of parallel slit cuts (the width about 0.1 mm) are obtained by laser cutting technology in the overall structure (the overall length of designed Type-I and Type-II SPIs are 79.2 and 61.2 mm, respectively). Both Type-I and Type-II SPIs are composed of 36 subcells, and the latter corresponds to 9 supercells. The detailed parameters are described in Results and Discussion. The metal layer of the SPI structure is made up of copper with 0.018-mm thickness. To make the overall manufacture process faster and more cost-efficient and reduce the effect of the heat-impacted zone on structure, we use a 15-W ultraviolet femtosecond laser cutting power and a marking speed of 900 μm/s, operating at a cutting frequency of 800 KHz. The cutting process is repeated 45 times. It is noted that the materials and metal thickness of the hybrid gradient plasmonic waveguide are the same as those of the SPI, but the thickness of the substrate is 0.508 mm.

### Simulation and measurement

Numerical simulations are performed by the CST Microwave Studio. For all simulated electromagnetic performances presented in this work, we neglect the influence of the substrate. The dispersion curves of the unit were calculated by using the eigenmode solver in the CST, and the boundary conditions have been taken as periodic in the *x* direction, and electrical boundaries (*E*_t_ = 0) in the *y* and *z* directions. Finite element analysis models are established in ABAQUS to study the mechanical behaviors of the deformable SPI. We use Agilent Vector Network Analyzer (Agilent 3656D) to measure all spectrum measurements including the transmission and reflection coefficients of the designed Type-I and Type-II samples. In addition, the near-field distributions are measured by a homemade near-electric-field scanning system (NFS03 Cabinet Version 3D), in which the probe is set as 3 mm above the deformable SPI samples. The force-displacement curves of different stretchable SPIs are measured through the universal testing machine.

Note: On-body epidermal experiment was performed on an author of this work, and the human subject involved in the experiments took part with informed consent. The research was noninvasive and harmless to humans, and no formal approval from institutional authorities was required.

## Data Availability

The data that support the findings of study are available from the corresponding authors upon reasonable request.

## Supplementary Materials

Supplementary 1 Sections S1 to S8 Figs. S1 to S19 Movies S1 and S2 Reference [54]
