# Supplementary material for: Kirigami-Triggered Spoof Plasmonic Interconnects for Radiofrequency Elastronics
Source: Research (Wash D C). 2024 May 1;7:0367. doi: 10.34133/research.0367 (PMC11062506; doi:10.34133/research.0367)
Supplement: Supplementary 1 — Sections S1 to S8 Figs. S1 to S19 Movies S1 and S2 Reference [54] [file research.0367.f1.zip › Revised supporting information (Clean Version).docx]

Supporting Information

**Kirigami-triggered Spoof Plasmonic Interconnects for Radiofrequency Elastronics**

Xincheng Yao1,2,†, Min Li1,2,†, Shuchang He3, Liqiao Jing1,*, Chenming Li1, Jie Tao1,2, Xiaonan Hui1, Fei Gao1,2, Jizhou Song3, Hongsheng Chen1,2,* and Zuojia Wang1,2,*

*1 State Key Laboratory of Extreme Photonics and Instrumentation, ZJU-Hangzhou Global Scientific and Technological Innovation Center, Zhejiang University, Hangzhou 310027, China.*

*2International Joint Innovation Center,* *Key Lab. of Advanced Micro/Nano Electronic Devices & Smart Systems of Zhejiang, The Electromagnetics Academy at Zhejiang University, Zhejiang University, Haining 314400, China*

*3Department of Engineering Mechanics, Key Laboratory of Soft Machines and Smart Devices of Zhejiang Province, State Key Laboratory of Brain-Machine Intelligence, Zhejiang University, Hangzhou 310027, China*

**Corresponding authors. E-mail:* [*liqiaoj@zju.edu.cn*](mailto:liqiaoj@zju.edu.cn) *(L. Jing);* [*hansomchen@zju.edu.cn*](mailto:hansomchen@zju.edu.cn) *(H. Chen);* [*zuojiawang@zju.edu.cn*](mailto:zuojiawang@zju.edu.cn) *(Z. Wang)*

*† These authors contributed equally to this work.*

**Supporting Information Guide:**

**-- Section S1. The effect of structure parameters on the dispersion curves**

-- **Section S2. The field distribution of the Type-I SPI**

-- **Section S3. The deformable process of designer SPIs**

-- **Section S4. The detailed parameters and electromagnetic performance of overall structure with hybrid plasmon waveguide and SPI**

-- **Section S5. The simulated transmission performance and finite element analysis of the deformable SPIs**

-- **Section S6. The electromagnetic performance of the stretchable SPIs**

-- **Section S7. The near-field scanning system and heartbeat monitoring setup**

**-- Section S8. Mechanical performance of the SPIs**

**Section 1. The effect of structure parameters on the dispersion curves.**

Figure S1 shows the dispersion curves of Type-I SPI that approach to different cutoff frequencies as the facet B length (*l*b) decreases from 3.0 to 1.0 mm, while ribbon width (*w*) is insensitive to that. Consequently, the surface plasmonic modes can be highly localized on the stretchable Type-I SPI surface, which exhibits strong field confinement, slow-wave dispersion characteristics and further excellent broadband transmission performance. Then combining two SPI sub-cells with different facet A length (*l*a) and ribbon widths (*w*1, *w*2) as shown in Figure 3a, a band-stop interconnect is obtained. The dispersion curves of the Type-II SPI with band-stop performance are shown in Figure S2 and S3. Obviously, although the width of the bandgap is insensitive to varied *l*b, it can increase as the *w* decreases. Here, we fix the difference between *w*1 and *w*2 as 0.5 mm.


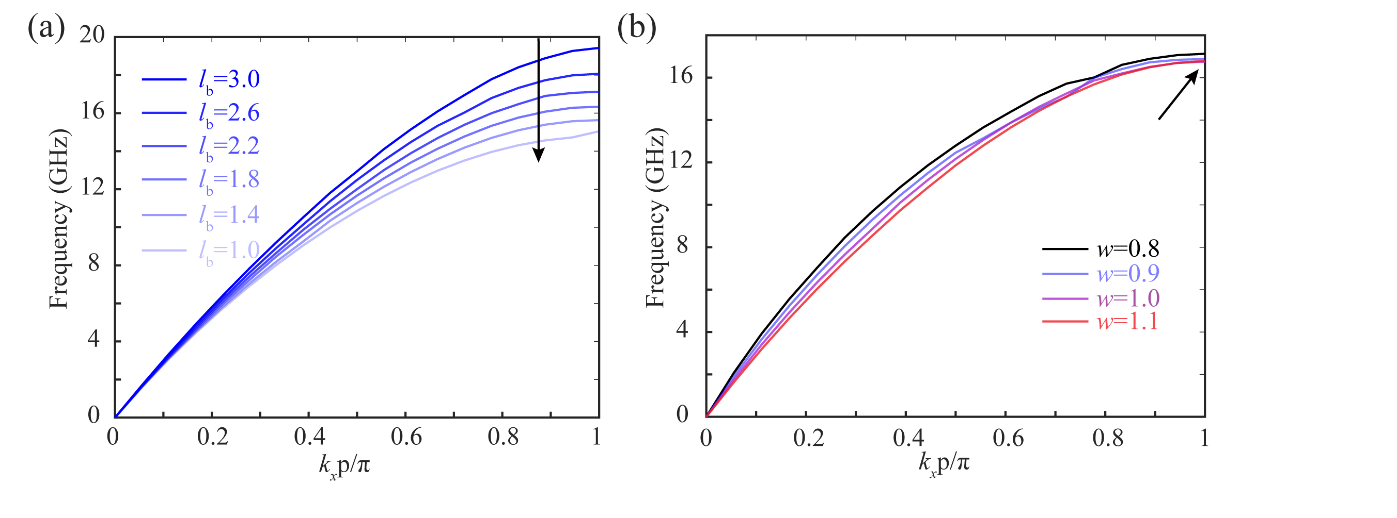


**Figure S1.** Dispersion curves as the structural parameters (a) *l*b and (b) *w* are varied for the Type-I SPI.


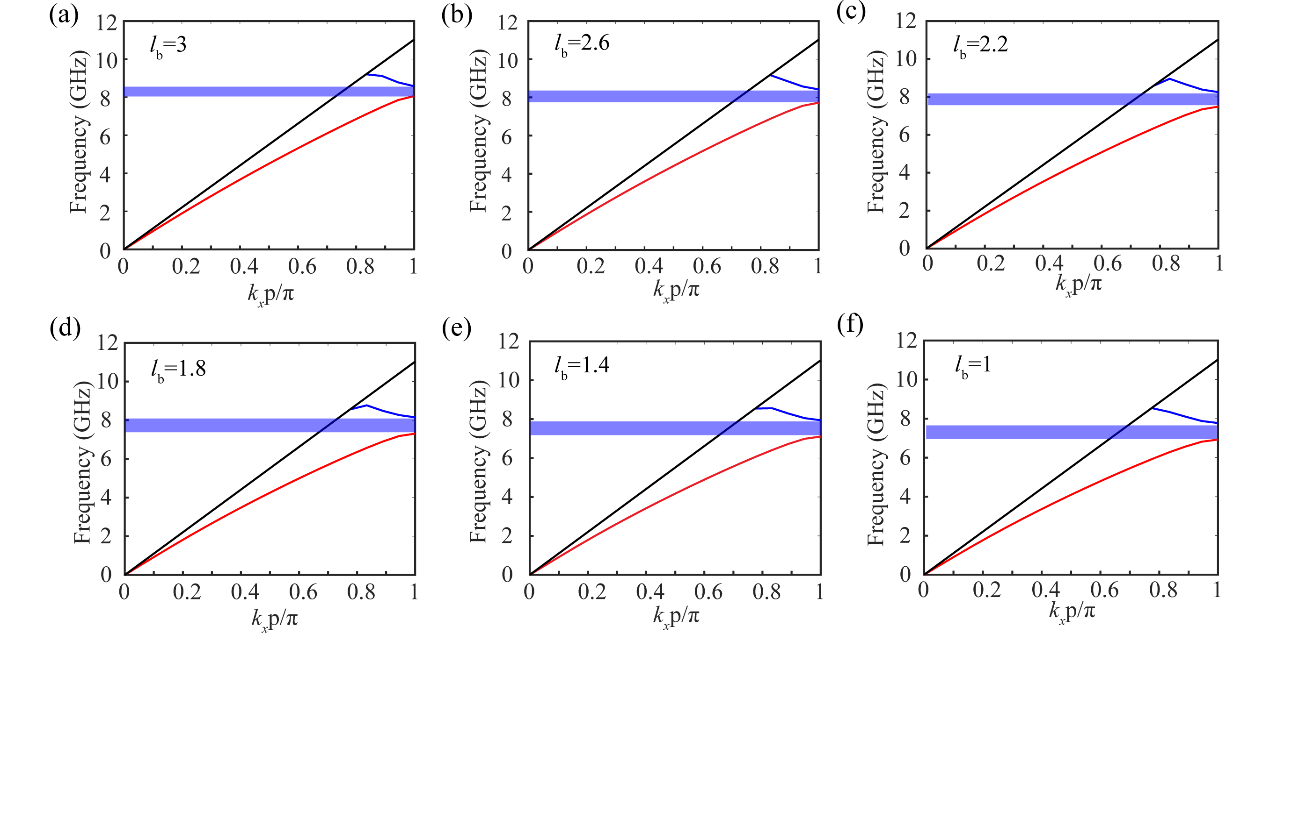


**Figure S2.** Dispersion curves of mode 1 (fundamental mode) and mode 2 (second mode) for the stretchable Type-II with different *l*b.


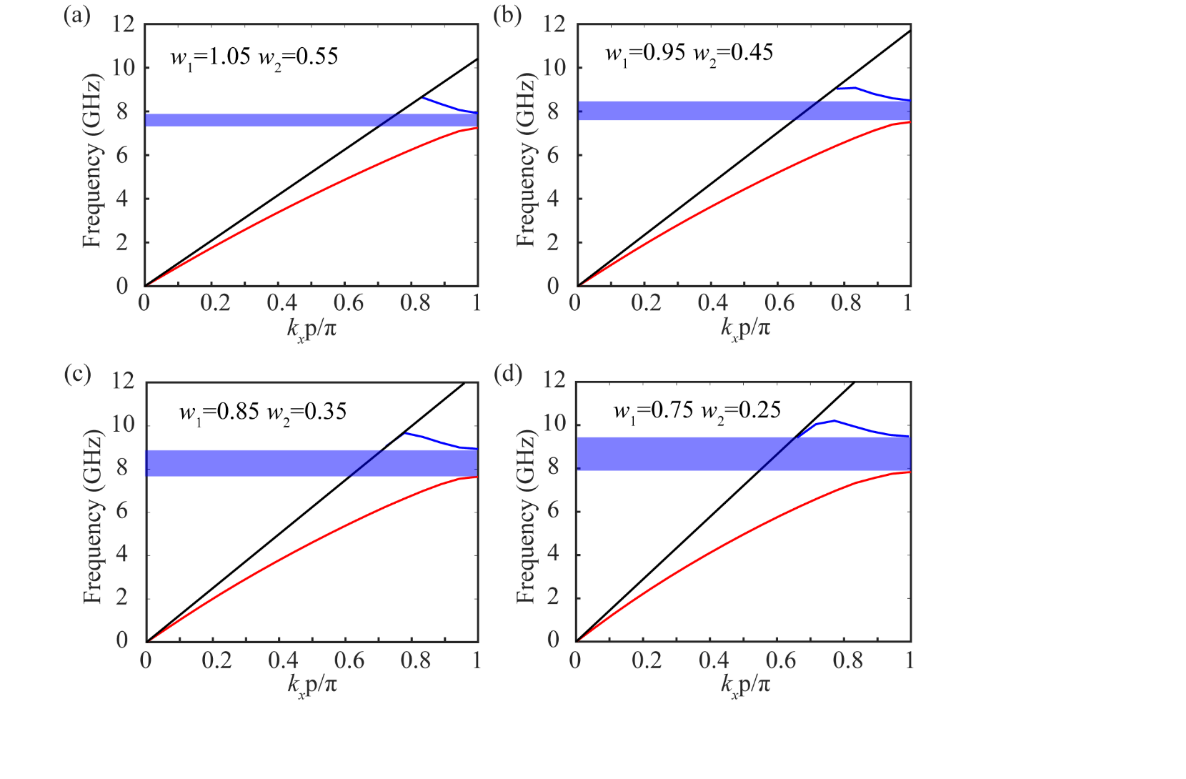


**Figure S3.** Dispersion curves of mode 1 and mode 2 for the stretchable Type-II with different *w*.

**Section 2. The field distribution of the Type-I SPI**


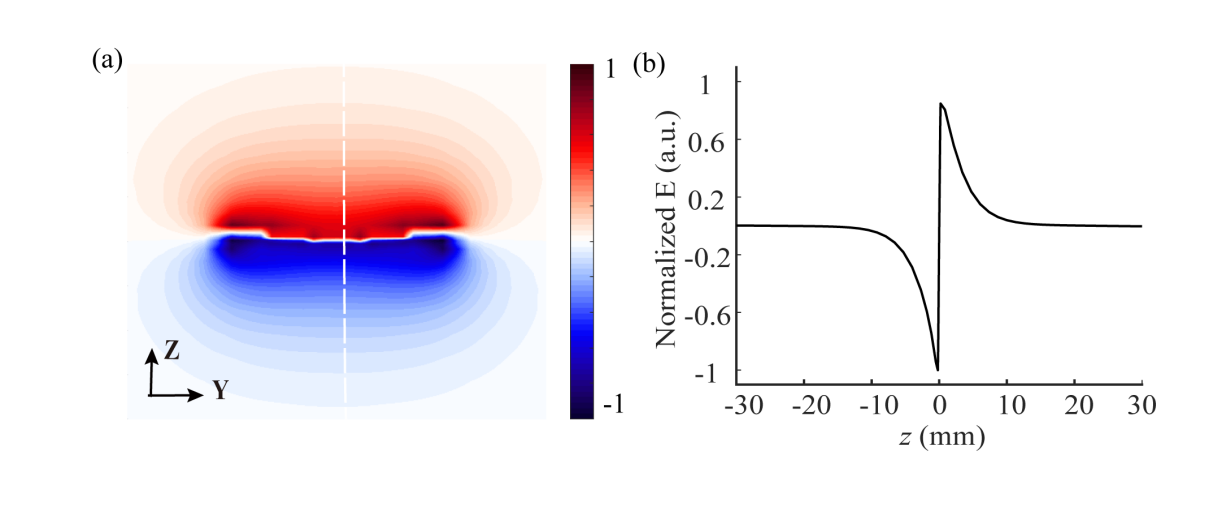


**Figure S4.** (a) Normalized field distribution on the *y*-*z* plane. (b) Field profile along the dashed line in the field distribution of (a).

**Section S3. The deformable process of designer SPIs**

For the proposed Type-I SPI, a few simple design parameters, i.e., the cut size *l*a = 4 mm, *l*b = 2 mm, *l*c = 5 mm, and the spacing between cuts *w* = 1 mm, can fully define the overall cutting pattern. When the kirigami sheet is stretched in-plane along the direction perpendicular to the cuts (the *x*−direction), internal stress concentrates on the cut tips, eventually forcing the facets to deform out-of-plane as the stretching reaches a special value[56]. With the stretchable values increasing after buckling, the rigid ribbon sheets will tilt accordingly. In practice, the tilting orientation of facets is single stability, dictated by pre-stretchable crease and loading conditions. As shown in Figure S5a and S5b, from a geometric point of view, when a Type-I SPI cell is in the initial state (without stretching), *θ* takes the minimum value (*θ* = 0°); when the buckled Type-I SPI cell is fully stretched state, the *θ* takes the maximum values (*θ*max= 90–tan–1(2*w*/*l*c)). The dihedral angle between facet C and the *x*-*z* reference plane is defined as *β* = cos–1(2*w*/*l*ctan(90–*θ*)). Similarly, when it is the flat state, *β* = 90°; when it is the fully stretched state, *β* = 0°, which reaches the geometrical limit of deformation[30]. And the facet A is always parallel to the facet B.


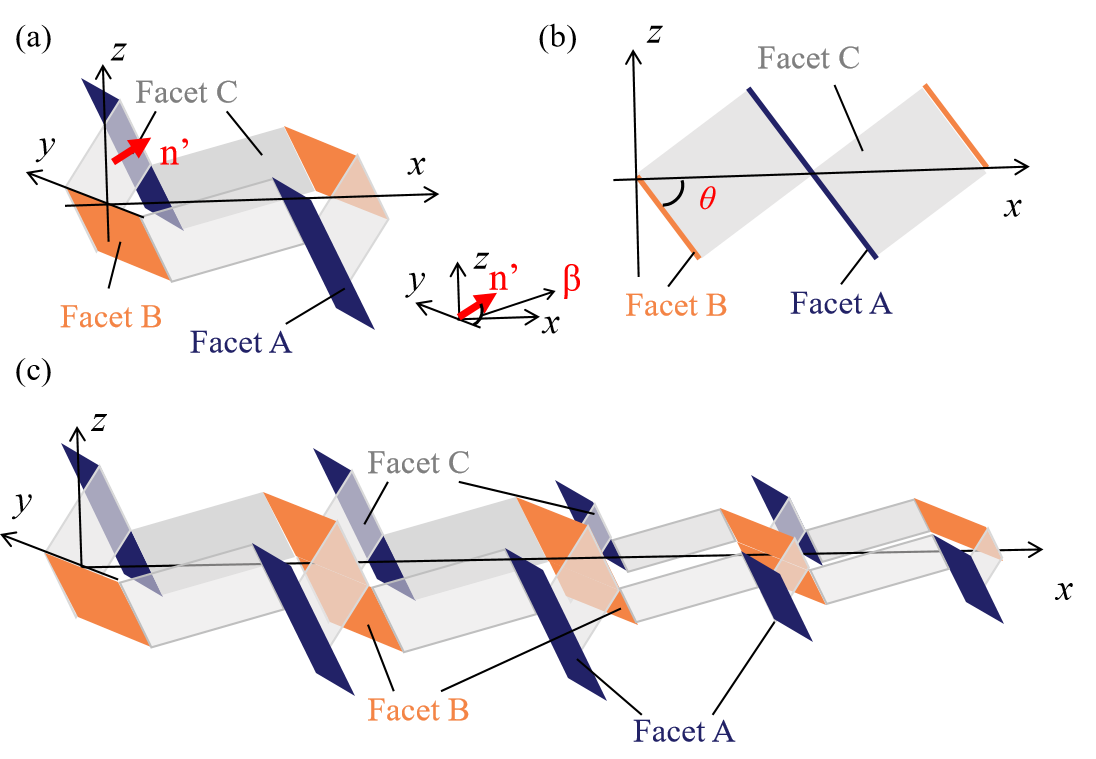


**Figure S5.** (a) The isometric and (b) side views of the stretchable Type-I cell. The red arrow (n’) indicates the normal vector perpendicular to the facet C. (c) The isometric view of the stretchable Type-II SPI cell.

There is the same deformable process of the proposed Type-II super-cell composed of large (i.e., the geography parameters of the Type-I SPI cell) and small sub-cell (*l*a = 2.7 mm, *l*b = 2 mm, *l*c = 5 mm, and *w* = 0.5 mm), comparing with that of the Type-I structure, as shown in Figure S5c. Importantly, assume a critical constraint regarding the direction of facet rotation based on the rigid facet: The adjacent facets’ tilted direction and rotation angle must be the same at the boundary between two different sub-cells for geography deformation.

**Section 4. The detailed parameters and electromagnetic performances of overall structure with hybrid plasmon waveguide and SPI**

To efficiently feed energy into and exact signals from the deformable SPI, we consider a hybrid plasmonic waveguide with a co-planar waveguide and matching transition as the excitation source. The whole structure is divided into three sections (A, B, and C), as shown in Figure S6a. Section A is a traditional co-planar waveguide with 50 Ω impedance (see Figure S6b), which supports the propagation of quasi-transverse electric and magnetic (TEM) mode. Region B is transition section composed of gradient grooves and flaring ground, overcoming the mismatch of impedance and mode between co-planar waveguide and SPI (Figure S6c)[38]. The curve of flaring ground is decribed as *y* = *C*1e*αx*+*C*2, where ,, α = 0.1, and (*x*1, *y*1) and (*x*2, *y*2) are the start and end points of the curve. The related design about section C is exhibited in the main text (Figure S6d).


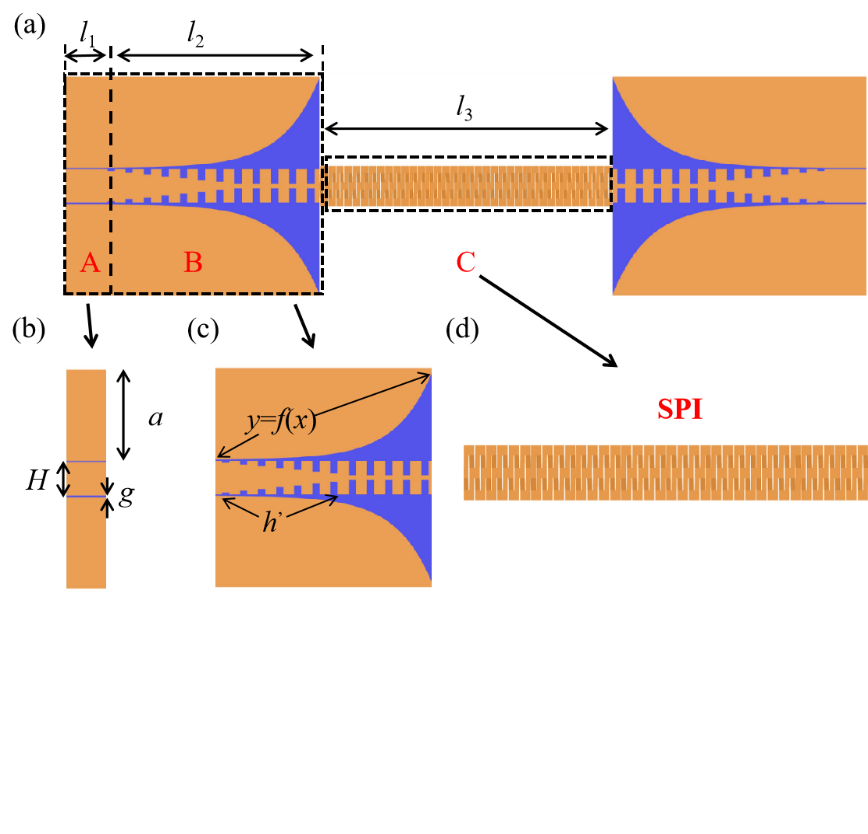


**Figure S6.** (a) The configuration of the overall structure with hybrid plasmonic waveguide (A and B) and SPI (C), in which *l*1 = 10 mm, *l*2 = 60 mm, *l*3 = 79.2 mm or 61.2 mm. (b) The co-planar waveguide section, in which *H* = 9.24 mm, *a* = 25 mm and *g* = 0.4 mm. (c) The matching transition with gradient grooves and flaring ground, in which *h’* = 0.5, 1, 1.5, 2, 2.5, 3, 3.5, 4 mm, and *y* = *f*(*x*) = C1e*αx*+C2 (*α* = 0.1). The substrate thickness is 0.508 mm. (d) The proposed SPI section.


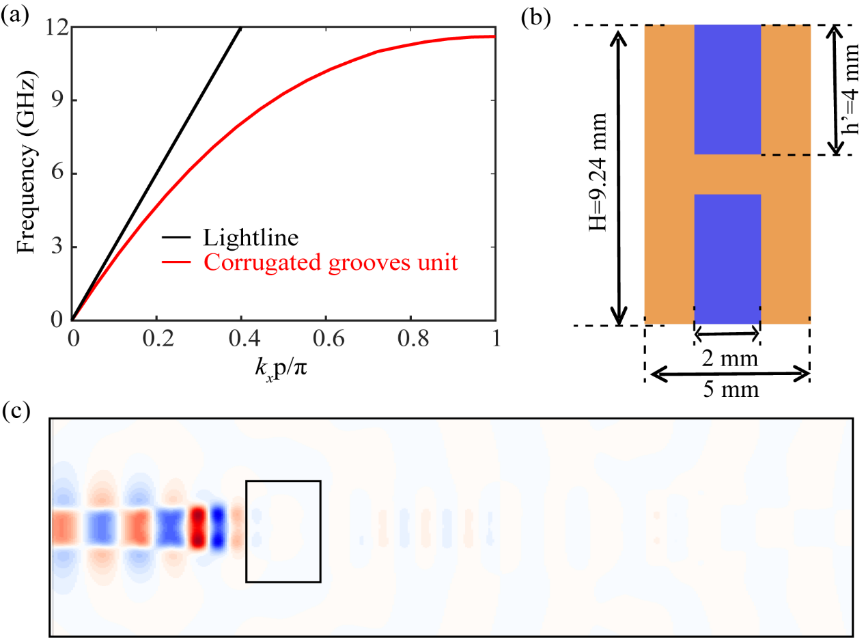


**Figure S7.** (a) Dispersion curves and (b) schematical structure of the corrugated grooves cell. (c) *E*z distribution of overall structure at 12 GHz. The black line shows the cell part of (b).

**Section 5. The simulated transmission performance and finite element analysis of the deformable SPIs**

The detailed geometric parameters of the deformable SPI are shown in Figure S6 and 2a. The materials used for the stretchable interconnects with hybrid plasmonic waveguide were Cu film and substrate layer (Rogers RT5880). The elastic modulus of Cu film and substrate are 119 GPa and 1.07 GPa, and the Poisson’s ratio are 0.34 and 0.3, respectively. As shown in Figure S10a, when subjected to a 15% in-plane uniaxial stretching, out-of-plane buckling deformation of the interconnect can naturally take place, leading to high stretchability. As expected, the principal strain distribution from FEA (Figure S10b and S10c) shows that the maximum principal strain in the stretchable SPI is concentrated at the tips of the slit cuts and relatively lower strain elsewhere. For the deformable SPI with bending and twisting, it is observed that the maximum principal strain is about 5.8% and 2.6% at the tips of the slit cuts, respectively, which is less than the damage limit (6%), indicating no breakage in the interconnect.


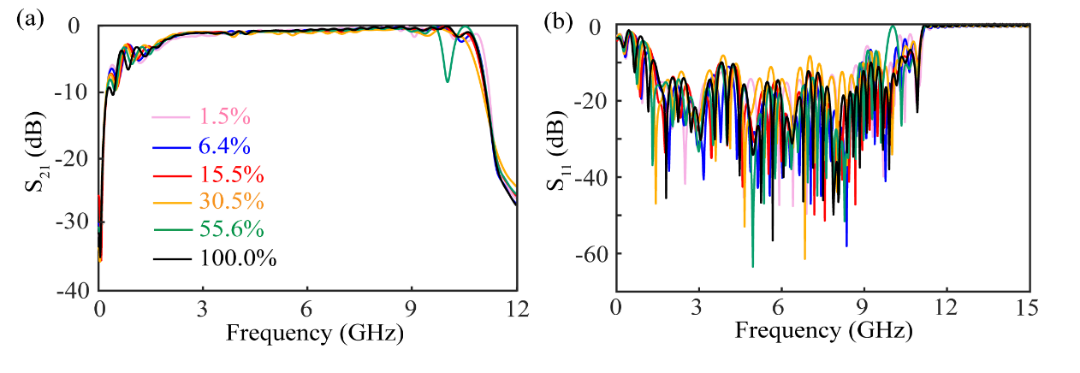


**Figure S8.** Simulated (a) transmission (S21) and (b) reflection (S11) efficiency of the Type-I SPI with different stretchable states.


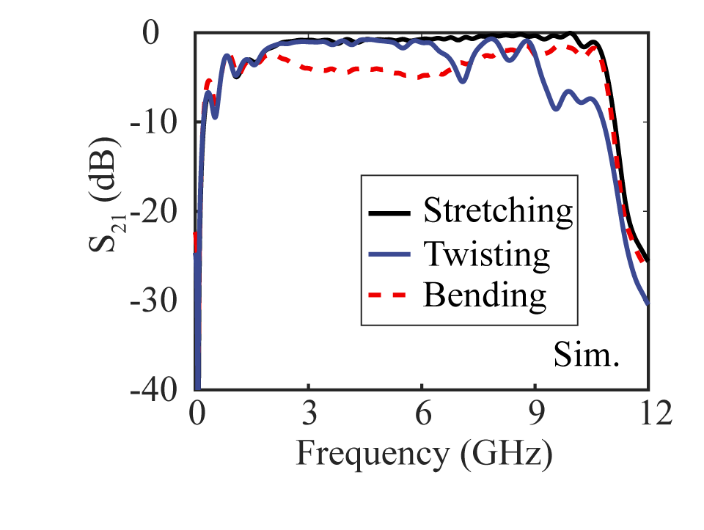


**Figure S9.** Simulated transmission performance of the Type-I SPI with different deformation structure.

**
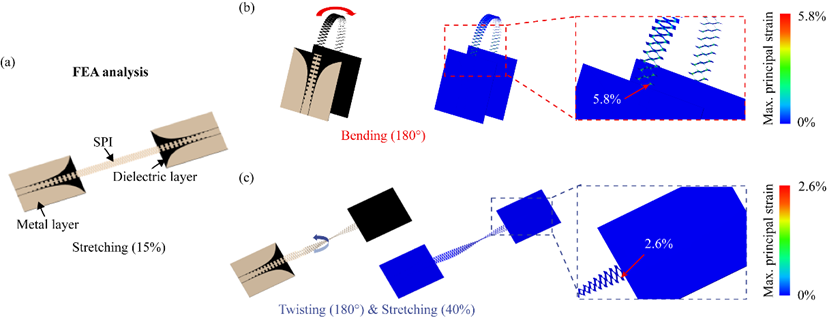
**

**Figure S10.** FEA simulation of the deformable SPI. (a) Schematics of the 15% stretching SPI. Maximum principal strain distribution of the SPI undergoing (b) 180° bending and (c) 180° twisting during stretching from 15% to 40%.

**Section 6. The electromagnetic performances of the stretchable SPI**


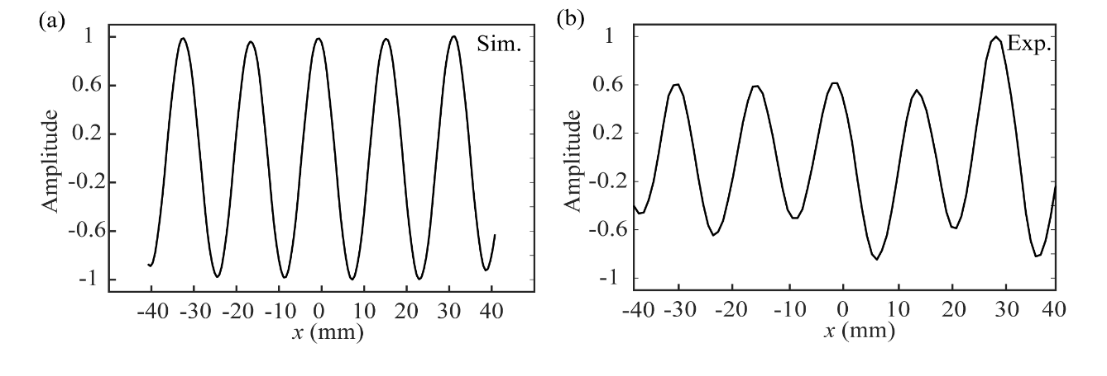


**Figure S11.** (a) Simulated and (b) measured normalized amplitude profiles along the middle line (*y* = 0) in *z*-component near-electric field distributions of Figure 2d.


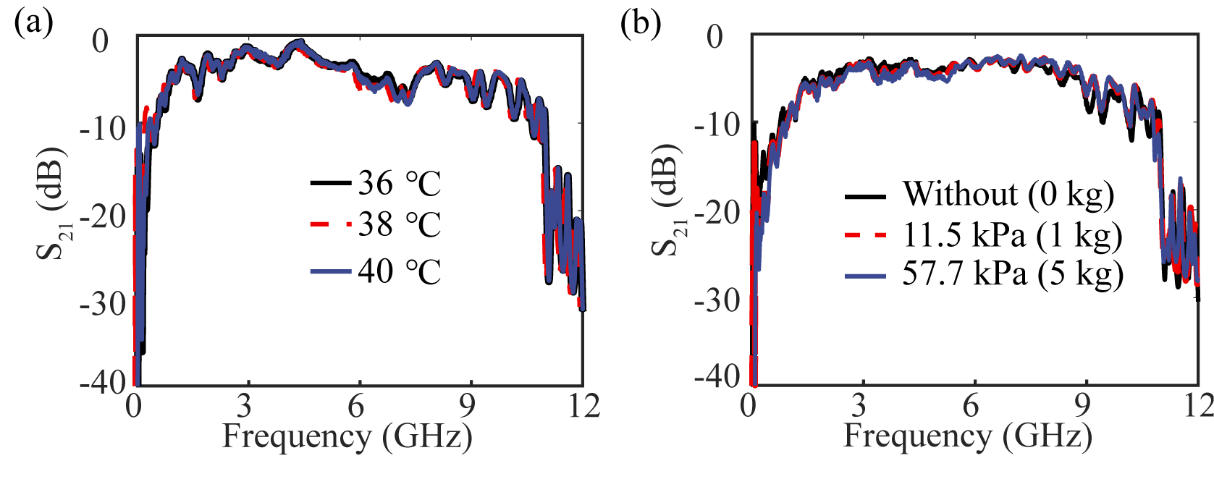


**Figure S12.** Measured transmission of the SPI with different temperatures and applied pressure.


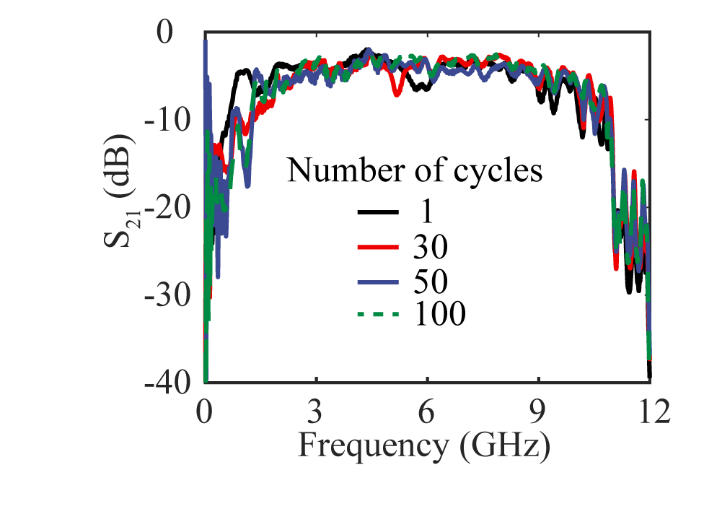


**Figure S13.** Repeatability transmission test of the interconnect to a cycling stretching over 100 cycles.


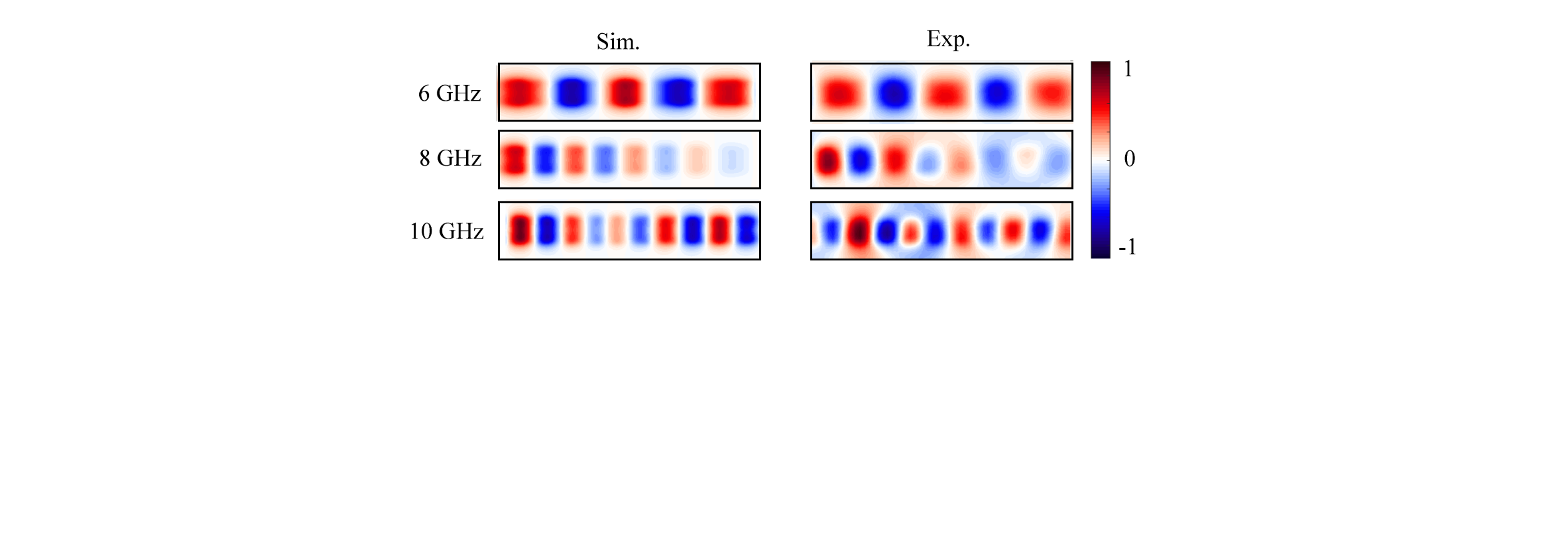


**Figure S14.** Simulated (left) and measured (right) *z*-component near-electric field distributions (on the plane 3 mm over the deformable Type-II SPI) of the Type-II SPI at 6, 8, and 10 GHz.


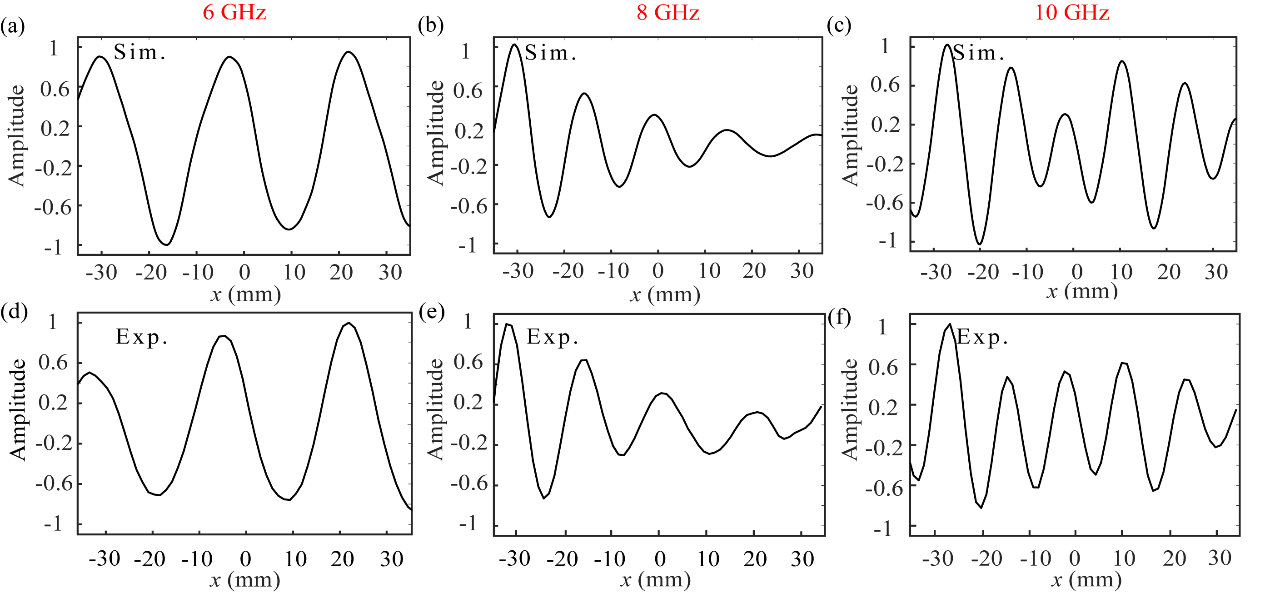


**Figure S15.** Normalized amplitude profiles of (a-c) simulation (upper) and (e-f) measurement (bottom) along the middle line (*y* = 0) of *z*-component near-electric field distributions at 6, 8, and 10 GHz.


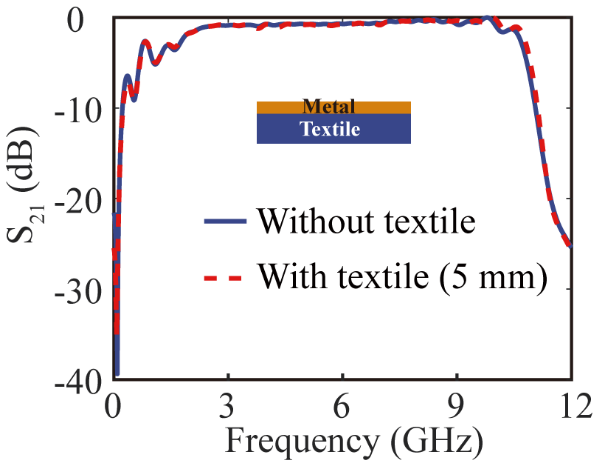


**Figure S16.** Simulated transmission of stretchable Type-I SPI with and without textile packaging (*ε*tex = 1.5 and the thickness of 5 mm). The inset shows that the adhesive metallic sheets are attached on textile.

**Section 7. The near-field scanning system and heartbeat monitoring setup**


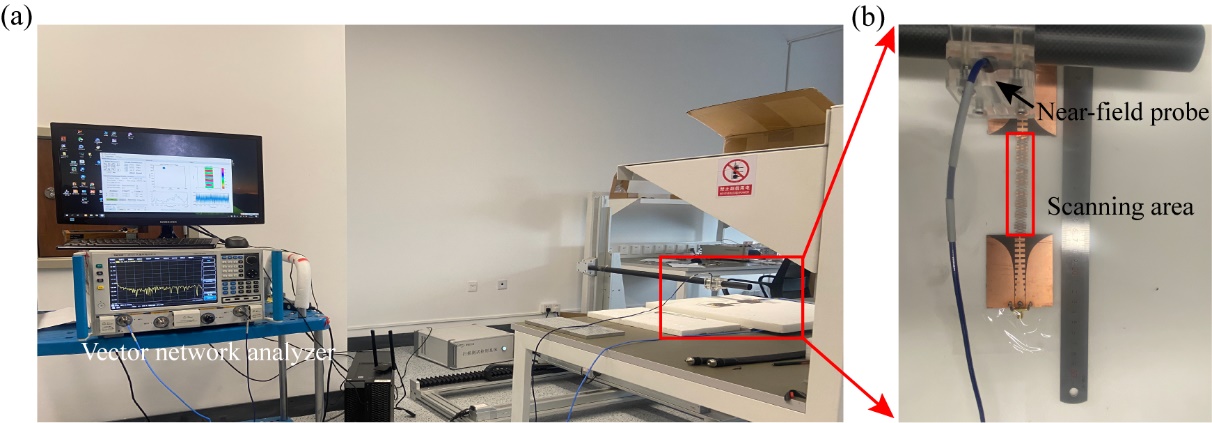


**Figure S17.** Homemade near-field experimental system.


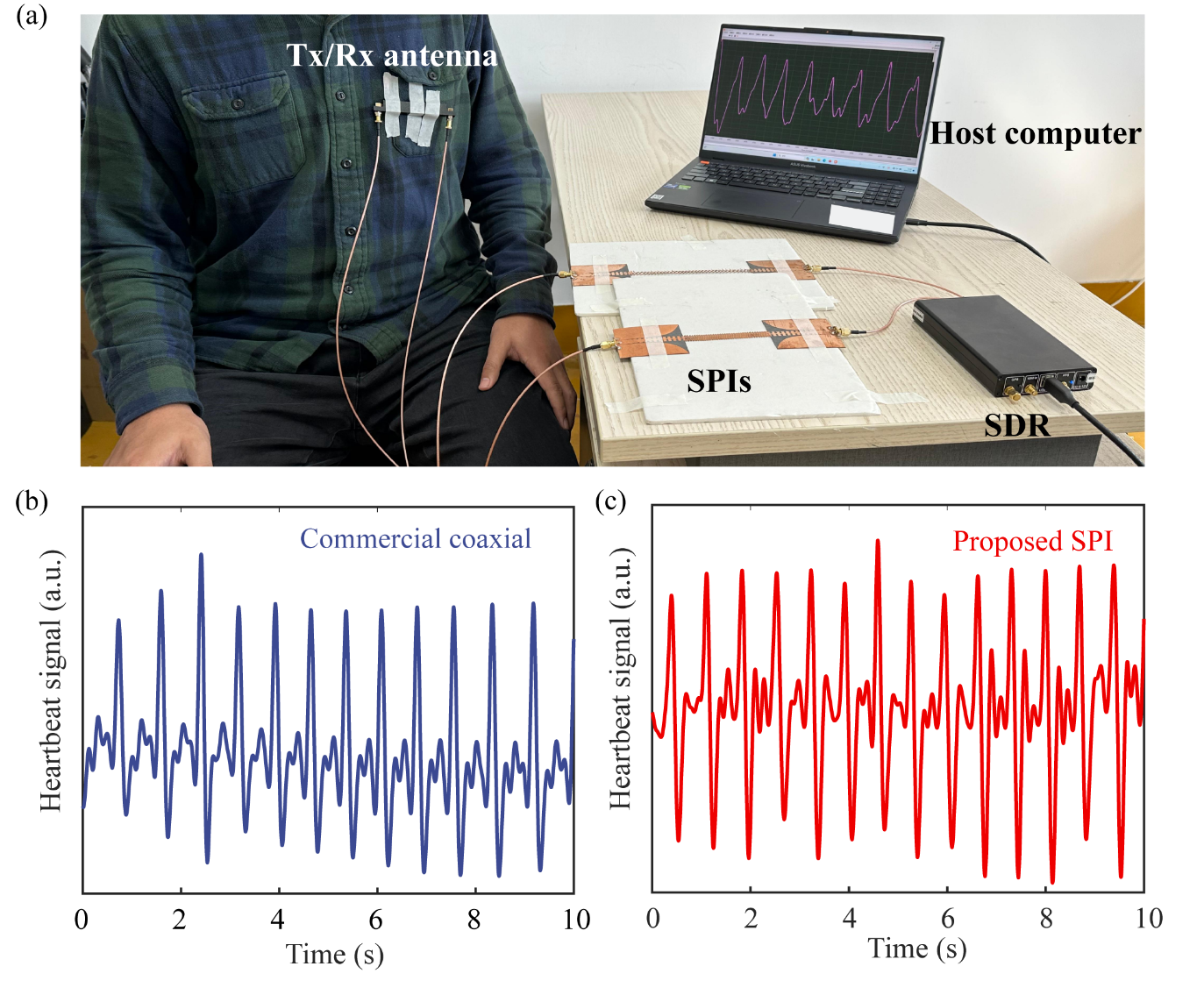


**Figure S18.** Experimental setup for the hearbeat dynamics extraction, where a Tx/Rx pair antenna is attached to the left chest of the subject.

**Section 8. Mechanical performance of SPIs**


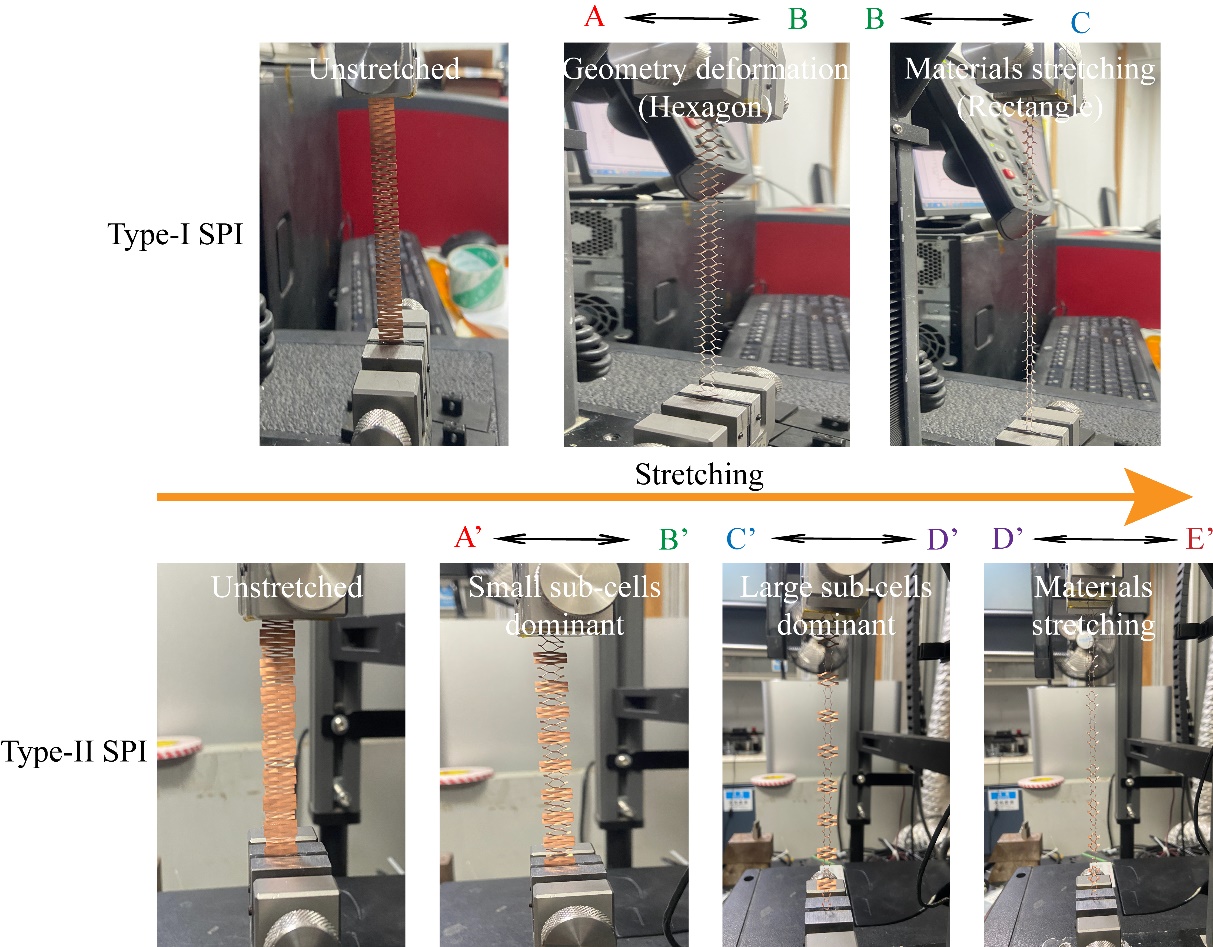


**Figure S19.** Mechanical performance of the SPIs subjected to uniaxial tension.
